# Effect of Non-Newtonian Flow on Polymer Flooding in Heavy Oil Reservoirs

**DOI:** 10.3390/polym10111225

**Published:** 2018-11-03

**Authors:** Xiankang Xin, Gaoming Yu, Zhangxin Chen, Keliu Wu, Xiaohu Dong, Zhouyuan Zhu

**Affiliations:** 1 College of Petroleum Engineering, China University of Petroleum, Beijing 102249, China; xiankang.xin@hotmail.com (X.X.); zhachen@ucalgary.ca (Z.C.); wukeliu19850109@163.com (K.W.); dongxh@cup.edu.cn (X.D.); zhuzy02@cup.edu.cn (Z.Z.); 2 College of Petroleum Engineering, Yangtze University, Wuhan 430100, China; 3 Department of Chemical and Petroleum Engineering, University of Calgary, Calgary, AB T2N 1N4, Canada

**Keywords:** polymer flooding, heavy oil, non-Newtonian flow, numerical simulation, threshold pressure gradient

## Abstract

The flow of polymer solution and heavy oil in porous media is critical for polymer flooding in heavy oil reservoirs because it significantly determines the polymer enhanced oil recovery (EOR) and polymer flooding efficiency in heavy oil reservoirs. In this paper, physical experiments and numerical simulations were both applied to investigate the flow of partially hydrolyzed polyacrylamide (HPAM) solution and heavy oil, and their effects on polymer flooding in heavy oil reservoirs. First, physical experiments determined the rheology of the polymer solution and heavy oil and their flow in porous media. Then, a new mathematical model was proposed, and an in-house three-dimensional (3D) two-phase polymer flooding simulator was designed considering the non-Newtonian flow. The designed simulator was validated by comparing its results with those obtained from commercial software and typical polymer flooding experiments. The developed simulator was further applied to investigate the non-Newtonian flow in polymer flooding. The experimental results demonstrated that the flow behavior index of the polymer solution is 0.3655, showing a shear thinning; and heavy oil is a type of Bingham fluid that overcomes a threshold pressure gradient (TPG) to flow in porous media. Furthermore, the validation of the designed simulator was confirmed to possess high accuracy and reliability. According to its simulation results, the decreases of 1.66% and 2.49% in oil recovery are caused by the difference between 0.18 and 1 in the polymer solution flow behavior indexes of the pure polymer flooding (PPF) and typical polymer flooding (TPF), respectively. Moreover, for heavy oil, considering a TPG of 20 times greater than its original value, the oil recoveries of PPF and TPF are reduced by 0.01% and 5.77%, respectively. Furthermore, the combined effect of shear thinning and a threshold pressure gradient results in a greater decrease in oil recovery, with 1.74% and 8.35% for PPF and TPF, respectively. Thus, the non-Newtonian flow has a hugely adverse impact on the performance of polymer flooding in heavy oil reservoirs.

## 1. Introduction

The worldwide demand for petroleum resources will continue to increase before new alternative energy sources are maturely developed and widely applied [1]. With this rapid growth in the demand of petroleum resources, heavy oil has attracted increasing attention because of its huge reserves [2,3]. Cold production techniques including water flooding, polymer flooding, surfactant flooding and alkaline flooding, as well as thermal recovery techniques including Huff and Puff, steam flooding, steam-assisted gravity drainage and in-situ combustion have been widely used for heavy oil exploitation [4]. Although a thermal recovery technology can reduce the viscosity of heavy oil to improve its recovery, it still suffers many disadvantages such as high costs and limited use in deep reservoirs [5]. Polymer flooding is one of the most mature technologies for improving the water flooding performance and enhanced oil recovery due to its ability in reducing the water-oil mobility ratio [6]. Moreover, polymer flooding can improve sweep efficiency in heterogeneous reservoirs compared with other cold recovery technologies [7]. Its successful application in heavy oil reservoirs has alleviated the pressure on the petroleum resources demand to some extent [8,9]. Therefore, polymer flooding is increasingly attracting attention in the development of heavy oil reservoirs [10].

Investigating fluid flow in porous media can provide theoretical guides for improving the performance of polymer flooding and enhanced oil recovery (EOR) in heavy oil reservoirs [11]. Many scholars have studied the polymer solution and heavy oil rheology and their flow in porous media [12,13,14]. It was found that the rheology of the polymer solution and heavy oil differs from that of Newtonian fluids, and exhibits non-Newtonian fluid characteristics because both polymer solution and heavy oil contain high molecular weight components, which form a spatial network structure [15,16]. Some researchers have combined rheological results from experiments and theories with their flow behaviors in porous media to enrich and improve the understanding of their transport in heavy oil reservoirs. For example, some models were proposed to convert a polymer solution flow rate into an equivalent shear rate to study its rheology in porous media [17,18,19,20], due to the fact that flow rate rather than the shear rate is generally known in the fluid flow through porous media [21]. In terms of heavy oil, it was reported that heavy oil needs to overcome a certain yield stress to flow, showing the characteristics of Bingham fluid [22,23]. A certain threshold pressure gradient (TPG), corresponding to this certain yield stress, must be overcome when heavy oil begins to flow in porous media [24,25]. The yield stress of heavy oil can be obtained by rheological experiments, while TPG can be obtained by TPG measurement experiments. Many methods including the steady pressure-velocity method [26], unstable method [27], capillary balance method [28], and micro-flow method [29] have been proposed to accurately measure TPG. Some of these methods have disadvantages such as large errors, are highly time-consuming, and have difficult data acquisition [30]. The micro-flow method is a recommended one because of its accuracy and acceptable time requirement [31]. The existing comprehensive flow research and accurate experimental measurements can provide a strong support for the EOR of polymer flooding in heavy oil reservoirs [32].

In addition, numerical reservoir simulation, a powerful approach, is a helpful complement to experiments in investigating the fluid flow in porous media and polymer flooding in heavy oil reservoirs. Compared with experiments, numerical reservoir simulation has advantages in efficiently predicting polymer flooding, as well as evaluating and analyzing factors controlling polymer flooding [33]. With the continuous improvement of numerical reservoir simulation, the numerical simulation of polymer flooding has made great progress, and can simulate most conventional fluid characteristics, flow mechanisms and processes [18,34]. Furthermore, the non-Newtonian flow of polymer solution can also be well illustrated [35]. Unlike the non-Newtonian flow of the polymer solution, the characteristics of the non-Newtonian flow of heavy oil, especially the threshold pressure gradient (TPG), are not well treated or considered in the numerical simulation of polymer flooding in heavy oil reservoirs [19]. Many commercial numerical simulators of polymer flooding such as ECLIPSE (Houston, TX, USA) and the Computer Modeling Group (CMG) STARS (Calgary, AB, Canada) have also had similar problems [36,37]. Although some methods have been proposed to describe TPG in the numerical simulation of heavy oil reservoir water flooding [38], the numerical simulation of polymer flooding in heavy oil reservoirs considering heavy oil TPG has rarely surfaced. This may lead to incorrect simulation results, and is unable to provide guidance of polymer flooding and its development forecasting in heavy oil reservoirs. Therefore, it is necessary to design a simulator that can accurately describe the heavy oil TPG for polymer flooding in heavy oil reservoirs.

In this paper, physical experiments were conducted to study the rheology of the polymer solution and heavy oil, and their flow in porous media. The viscosities of a polymer solution with the same polymer concentration and an original heavy oil were measured at different shear rates to recognize their rheology, and the viscosities of polymer solutions with different polymer concentrations were measured to estimate the increasing viscosity capacity of the polymer. The micro-flow method was applied to measure the heavy oil TPGs, and a typical polymer flooding (TPF) experiment was carried out to analyze the heavy oil flow in porous media. In addition, a new mathematical model was proposed, and an in-house 3D two-phase simulator was designed with consideration of heavy oil TPG for polymer flooding in heavy oil reservoirs. The designed simulator was validated by comparing its simulation results with those from the ECLIPSE V2013.1 software (Houston, TX, USA) and the TPF experiments. Moreover, the designed simulator was applied to investigate the effects of non-Newtonian flow on production indicators, including a pressure difference, water cut, and oil recovery. These results can assist to improve the performance of polymer flooding in heavy oil reservoirs.

## 2. Methodology

### 2.1. Physical Experiments

#### 2.1.1. Materials

**Figure 1:**
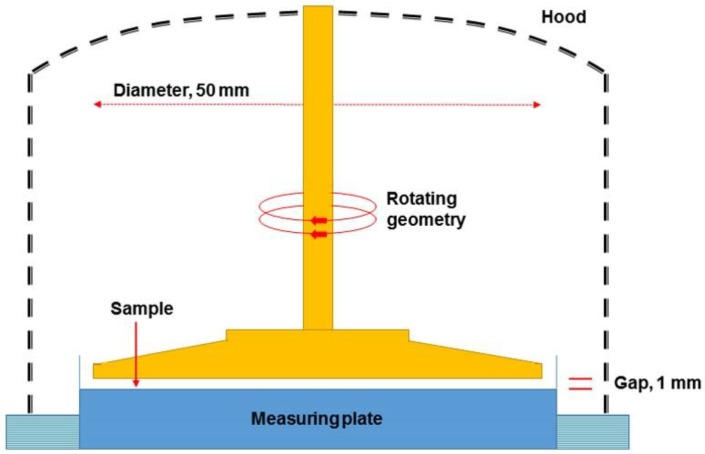
Schematic diagram of the rheometer with parallel-plate geometry.

The information of the materials including the polymer, brine, original heavy oil, and core samples are provided in Table 1, Table 2, Table 3, Table 4 and Table 5, respectively. Here, the SARA (saturates, aromatics, resins and asphaltenes) fractions and viscosity of the original heavy oil (marked as heavy oil sample #1) were measured on a CG-CF11 rod-thin-layer chromatography from Chuange Sence (Changsha, China) and a Physica MCR301 advanced rotary rheometer from Anton Paar (North Ryde, NSW, Australia). The schematic diagram of the rheometer with a parallel-plate geometry is presented in Figure 1, and the measurement gap was set to 1 mm for all heavy oil and polymer solution samples in this paper. The heavy oil samples #2, #3 and #4 with viscosities of 162.2, 118.7, and 73.8 mPa·s, respectively, at 25 °C, as used in this paper, were reconstituted oil obtained by mixing the original heavy oil with kerosene. The core samples #1–4 were obtained from the same large artificial core as well as the core samples #5–8, #9–12 and #13–16, so they were considered to have the same properties although their measured permeability and porosity are slightly different.

#### 2.1.2. Polymer Solution Preparation

The polymer solution was prepared at an ambient temperature of 25 °C, and its procedure was as follows:

1. One-hundred-and-ninety-nine milliliters of brine, whose ion component concentrations are shown in Table 2, was stirred under 200 revolutions per minute (rpm) using a JJ-1B stirrer from Xinrui Instrument Factory (Changzhou, China).
2. 1.096 g of polymer, whose properties are given in Table 1, was evenly added to the brine vortex in 30 s.
3. The stirrer speed of the JJ-1B stirrer was reduced to 100 rpm, and continued to run for 2 h.
4. The JJ-1B stirrer was stopped, and the polymer solution was deoxidized, sealed and statically stored in a brown glass bottle for 12 h.
5. Steps 1–4 were repeated, and 400 mL of polymer solution with a concentration of 5000 mg/L was obtained.
6. Fifty milliliters of polymer solutions with a concentration of 5000 mg/L were diluted with 450, 200,116.67, 50 mL of brine, and 200 mL of polymer solution with a concentration of 5000 mg/L was diluted with 300 mL of brine.
7. After dilution, all polymer solutions were stirred at 100 rpm for 0.5 h using the JJ-1B stirrer.
8. The JJ-1B stirrer was stopped. To simulate the polymer degradation caused by a high shear rate in a near-wellbore region, all polymer solutions were sheared under 16,900 rpm for 35 s using a Waring 7012S blender (Waring Products, Torrington, CT, USA) to perform pre-shearing.
9. The Waring 7012S blender was stopped, and all polymer solutions were deoxidized, sealed, and statically stored in brown glass bottles for 12 h. Five-hundred, 250, 166.67, 500 and 100 mL of polymer solutions with concentrations of 500, 1000, 1500, 2000, and 2500 mg/L, respectively, were obtained.

#### 2.1.3. Rheological Testing

The rheological testing analyzed the polymer solution with a concentration of 2000 mg/L and heavy oil sample #1. The viscosity of the polymer solution with different concentrations was tested at a shear rate of 7.6 s^−1^ and the tests were performed on the Physica MCR301 advanced rotary rheometer at 25 °C.

#### 2.1.4. Threshold Pressure Gradient Measurement

**Figure 2:**
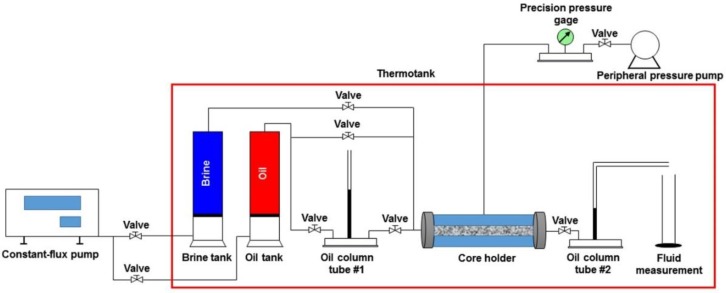
The schematic of a TPG measurement.

The flow chart in Figure 2 shows the order of devices used for TPG measurement. The procedures are as follows:

1. The experimental devices were connected according to Figure 2, and the temperature of the thermotank was set to 25 °C.
2. The brine was used to displace the core sample at a flow rate of 0.05 mL/min until the volume of injected heavy oil sample reached four times the pore volume (PV) of the core sample, which had been saturated with the brine and placed for at least 24 h before the TPG measurement started.
3. The heavy oil sample was used to displace the brine in the core sample at a flow rate of 0.05 mL/min after bypassing the oil column tube #1. The displacing flow rate was increased to 0.5 mL/min when the water cut at the outlet was lower than 2% until no water production. The core sample was saturated with bound brine and a heavy oil sample was obtained.
4. The height of the oil column in the oil column tube #2 was lowered to about 5 cm, and the oil column in tube #1 was put into use. The height of the oil column in oil column tube #1 was raised to the same height as that in oil column tube #2. This condition was kept for 24 h.
5. The heavy oil sample was used again to displace with a flow rate of 0.002 mL/min, and the height of the oil column in the oil column tube #1 increased gradually. The pressure gradient was the TPG of the heavy oil sample when the height of the oil column in oil column tube #2 began to rise.

Notably, air was prevented from entering the measurement system to avoid its effect on the accuracy of the TPF measurement.

#### 2.1.5. Typical Polymer Flooding Experiments

**Figure 3:**
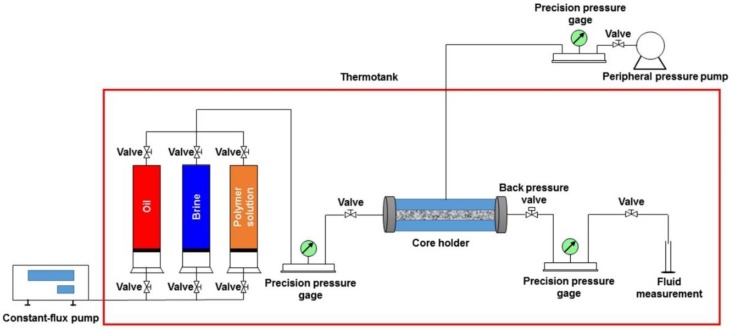
The schematic of a typical polymer flooding experiment.

The flow charge of a typical polymer flooding (TPF) experiment is presented in Figure 3, and its procedure is as follow:

1. The experimental devices were connected according to the flow chart, and the temperature of the thermotank was also set to 25 °C.
2. The process of completely saturating a core sample with a heavy oil sample and bound brine was similar to that of the TPG measurement.
3. Initial water flooding. After saturating, the brine was used to displace at a constant flow rate of 0.4 mL/min until the volume of the injected brine reached 1 PV.
4. Polymer flooding. The polymer solution with a concentration of 5000 mg/L was used to displace at a constant flow rate of 0.4 mL/min until the volume of the injected brine reached 0.4 PV.
5. Extended water flooding. The brine was used again to displace at a constant flow rate of 0.4 mL/min until the volume of the injected brine reached 2.6 PV.

Notably, avoiding air was also required during the TPF experiments.

### 2.2. Mathematical Model

#### 2.2.1. Assumptions

The assumptions for the mathematical model included:

1. Only the oil and water phases were involved in the polymer flooding process and no mass exchange occurred between them.
2. The mixture of water and polymer was ideal, and was presented only in the water phase.
3. The fluids were compressible, and the rock was not only compressible but anisotropic.
4. The flow was isothermal, and non-Newtonian flow was advised.
5. The injection of polymer only affected the permeability of the water phase and did not affect the permeability of the oil phase.
6. The effects of the capillary force and gravity were taken into account.

#### 2.2.2. Mechanisms

Compared to water flooding, the polymer flooding process is more complicated and is accompanied by complex parameter changes. This is especially true for polymer flooding in heavy oil reservoirs. More parameter changes need to be considered and mainly include the viscosity changes of the water phase, polymer adsorption, the permeability reduction of the water phase, inaccessible pore volumes, and the TPG of heavy oil.

The viscosity of the water phase changes with the injection of the polymer solution and is affected by the mixing degree of the water and the injected polymer solution, polymer concentration, and a shear rate. First, the Todd-Longstaff mixing parameter was introduced to describe the mixing degree of the water and the injected polymer solution [39,40]. In this study, the mixing of the water and the injected polymer solution was ideal, and the Todd-Longstaff mixing parameter was set to one, which meant that the effective viscosity of the water phase was equal to that of the fully mixed polymer solution. Moreover, the Flory–Huggins equation was applied to treat the relationship between the viscosity of the polymer solution at the zero shear rate and the concentration of the polymer and salt, which was written as [18,20,41]:

(1) $$\mu_{p}^{0}=\mu_{w}\left[1+\left(a_{p1}c_{p}+a_{p2}c_{p}^{2}+a_{p3}c_{p}^{3}\right){c_{s}}^{s_{p}}\right]$$

where $\mu_{p}^{0}$ is the viscosity of the polymer solution at the zero shear rate in Pa·s, $\mu_{w}$ is the water viscosity in Pa·s, $a_{p1},$ $a_{p2}$ and $a_{p3}$ are the parameters in (kg/m^3^)^−1^; $c_{p}$ is the polymer concentration in kg/m^3^; $c_{s}$ is the salt concentration in kg/m^3^; $s_{p}$ is the slope between $\left(\mu_{p}^{0}-\mu_{w}\right)/\mu_{w}$ and $c_{p}$ on a log-log plot. Here, the effect of the salt concentration was neglected by setting $s_{p}$ to zero. In addition, the dependence of the polymer solution viscosity on shear rate was modeled by Meter’s equation, which is expressed as [19,42]

(2) $$\mu_{ps}=\mu_{w}+\frac{\mu_{p}^{0}-\mu_{w}}{1+{\left(\frac{{\dot{\gamma }}_{eq}}{{\dot{\gamma }}_{1/2}}\right)}^{\left(a_{p4}-1\right)}}$$

where $\mu_{ps}$ is the shear viscosity of the polymer solution in Pa·s. ${\dot{\gamma }}_{eq}$ is the equivalent shear rate in s^−1^, ${\dot{\gamma }}_{1/2}$ is the shear rate at which the viscosity is equal to $\frac{\left(\mu_{p}^{0}+\mu_{w}\right)}{2},$ and $a_{p4}$ is a parameter. Here, Equation (3) was proposed to describe the relationship between the equivalent shear rate and the Darcy velocity of the polymer solution [17].

(3) $${\dot{\gamma }}_{eq}=4{\left(\frac{3n+1}{4n}\right)}^{\frac{n}{n-1}}\frac{v_{p}}{\sqrt{8k\phi \sigma }}$$

where n is the flow behavior index, $v_{p}$ is the Darcy velocity of the polymer solution in m/s, and k is the permeability of rock in m^2^. ϕ is the porosity, and σ is the tortuosity of pores. Here σ is equal to 25/12.

During the flow of polymer solution through the rock, some polymer molecules will inevitably be adsorbed on the inner surfaces of pores in the rock [43]. Here, the Langmuir adsorption isotherm model was applied to treat polymer adsorption [44,45]:

(4) $$c_{ap}=c_{apmax}\frac{b_{p}c_{p}}{1+b_{p}c_{p}}$$

where $c_{ap}$ is the adsorbed concentration of polymer in kg/kg, $c_{apmax}$ is the maximum adsorbed concentration of polymer in kg/kg, and $b_{p}$ is the adsorption coefficient.

The adsorbed polymer molecules will cause a permeability reduction of the water phase [18]. Here, the permeability reduction of the water phase was implemented by a factor of $R_{k}$, which is [46]:

(5) $$R_{k}=1+\left(\mathrm{RRF}-1\right)\frac{c_{ap}}{c_{apmax}}$$

where RRF is the residual resistance factor, defined as the ratio between the brine permeability measured before and after the polymer solution flows through the core.

Polymer cannot enter some small pores when the polymer solution flows through the rock, which results in an inaccessible pore volume [47]. Here, the inaccessible pore volume is treated by a factor of $f_{ipv}$, which is given as:

(6) $$f_{ipv}=\frac{V_{i}}{V_{p}}$$

where $V_{i}$ is the polymer inaccessible pore volume in m^3^ and $V_{p}$ is the pore volume in m^3^.

Heavy oil needs to overcome a TPG to flow [48], and the TPG of heavy oil can be calculated from the TPG measurement experiments.

#### 2.2.3. Equations

The flow of the water phase was in accordance with Darcy’s law, and its flow equation was [20]:

(7) $$\vec{\nu_{w}}=\frac{\vec{k}k_{rw}}{\mu_{wp}R_{k}}\nabla \Phi_{w}$$

where $\vec{\nu_{w}}$ is the water phase velocity tensor in m/s, $\vec{k}$ is the absolute permeability tensor in m^2^, $k_{rw}$ is the relative permeability of brine, and $\mu_{wp}$ is the viscosity of the water phase in Pa·s; ∇ is the gradient operator, and $\Phi_{w}=p_{w}-\rho_{w}gD$ in Pa; $p_{w}$ is the pressure of the water phase in Pa, $\rho_{w}$ is the density of the water phase in kg/m^3^, g is the gravitational acceleration in m/s^2^, and D is the vertical height of m.

Because the heavy oil flow through the rock no longer follows Darcy’s law, the oil phase flow equation had to be corrected with consideration of the TPG of heavy oil [24]:

(8) $$\vec{\nu_{o}}=\{\begin{array}{c} \frac{\vec{k}k_{ro}}{\mu_{o}}\left(\nabla \Phi_{o}-G\right)\;if\;\nabla \Phi_{o}>G\\ 0\;if\;\nabla \Phi_{o}\leq G \end{array}$$

where $\vec{\nu_{o}}$ is the oil phase velocity tensor in m/s, $k_{ro}$ is the relative permeability of the oil phase, and $\mu_{o}$ is the viscosity of the oil phase in Pa·s; $\Phi_{o}=p_{o}-\rho_{o}gD$ in Pa, $p_{o}$ is the pressure of the oil phase in Pa, $\rho_{o}$ is the density of the oil phase in kg/m^3^, and G is the TPG of the oil phase in Pa/m.

According to the conservation of mass, the continuity equations of all components in ground standard conditions can be obtained by combing the flow equations:

for water:
  (9) $$\nabla \cdot \left(\frac{\vec{\nu_{w}}}{B_{w}}\right)+q_{w}=\frac{\partial }{\partial t}\left(\frac{\phi s_{w}}{B_{w}}\right)$$
for polymer:
  (10) $$\nabla \cdot \left(\frac{\vec{\nu_{w}}}{B_{w}}\right)+q_{w}c_{p}=\frac{\partial }{\partial t}\left[\frac{\phi \left(1-f_{ipv}\right)s_{w}c_{p}}{B_{w}}\right]+\frac{\partial \left[\left(1-f_{ipv}\right)\left(1-\phi \right)\rho_{r}c_{ap}\right]}{\partial t}$$
for oil:
  (11) $$\nabla \cdot \left(\frac{\vec{\nu_{o}}}{B_{o}}\right)+q_{o}=\frac{\partial }{\partial t}\left(\frac{\phi s_{o}}{B_{o}}\right)$$

where $B_{w}$ and $B_{o}$ are the water and oil phase formation volume factors in m^3^/m^3^, respectively. $q_{w}$ and $q_{o}$ are the source/sink terms for the water and oil phases in m^3^/(s·m^3^), respectively. The source term is negative, and the sink term is positive. ∂ is the symbol used to denote partial derivatives, t is time in s; $s_{w}$ and $s_{o}$ are the water and oil phase saturations, respectively; $\rho_{r}$ is the rock density in kg/m^3^.

Although the flow and continuity equations described the basic flow characteristics, the interrelationship between some physical quantities in the equations needed to be additionally described by the auxiliary equation and the equations of state.

The auxiliary equations included:

(12) $$s_{w}+s_{o}=1$$

(13) $$p_{cow}\left(s_{w}\right)=p_{o}-p_{w}$$

where $p_{cow}\left(s_{w}\right)$ is the capillary pressure in the water-oil system in Pa, which is a function of the water phase saturation.

The equations of fluids, rock and rock-fluids included:

(14) $$k_{ro}=k_{ro}\left(s_{w}\right)$$

(15) $$k_{rw}=k_{rw}\left(s_{w}\right)$$

(16) $$\rho_{o}=\rho_{o}\left(p_{o}\right)$$

(17) $$\rho_{w}=\rho_{w}\left(p_{w}\right)$$

(18) $$\phi =\phi \left(p_{r}\right)$$

where $p_{r}$ is the reservoir pressure in Pa.

#### 2.2.4. Solution Method

The above equations provided a general description of polymer flooding in heavy oil reservoirs and might have multiple solutions. Therefore, definite conditions were given to obtain the unique solution for this specific study. The definite conditions included the initial and boundary conditions.

The initial conditions included the distribution of initial pressure, saturation and polymer concentration:

(19) $$p_{r}\left(x,y,z\right){|}_{t=0}=p_{ri}\left(x,y,z\right)$$

(20) $$s_{w}\left(x,y,z\right){|}_{t=0}=s_{wi}\left(x,y,z\right)$$

(21) $$c_{p}\left(x,y,z\right){|}_{t=0}=c_{pi}\left(x,y,z\right)$$

where (x,y,z) are the coordinates, $p_{ri}$ is the initial reservoir pressure in Pa, $s_{wi}$ is the initial water phase saturation, and $c_{pi}$ is the initial polymer concentration in kg/m^3^.

The boundary conditions included the outer and inner boundaries. The outer boundary was a closed boundary with no-flow, which was:

(22) $${\frac{\partial p}{\partial n}|}_{B}=f\left(x,y,z,t\right)=0$$

where ${\frac{\partial p}{\partial n}|}_{B}$ denotes the derivative of the boundary pressure in the direction of the outer normal. The inner boundary conditions were calculated as follows:

(23) $$Q_{w}\left(x,y,z,t\right){|}_{{\left(x,y,z\right)}_{well}}=Q_{w}\left(t\right)$$

(24) $$Q_{o}\left(x,y,z,t\right){|}_{{\left(x,y,z\right)}_{well}}=Q_{o}\left(t\right)$$

(25) $$c_{p}\left(x,y,z,t\right){|}_{{\left(x,y,z\right)}_{well}}=c_{p}\left(t\right)$$

where $Q_{w}$ and $Q_{o}$ are the flow rates of the water and oil phases, respectively; ${\left(x,y,z\right)}_{well}$ is the grid coordinate of a well.

Although the definite conditions were given, it was very difficult to solve the above partial differential equations by analytical methods [49], especially for the 3D model. Therefore, the control volume finite difference method, a numerical method, was proposed to solve the equations, and a block-centered grid was simultaneously employed as the grid system. The corresponding discretized forms of the continuity equations are as follows:

for water:
  (26) $$\begin{array}{l} {\left(T\lambda_{w}\Delta \Phi_{w}\right)}_{i+1/2,j,k}^{n+1}-{\left(T\lambda_{w}\Delta \Phi_{w}\right)}_{i-1/2,j,k}^{n+1}+{\left(T\lambda_{w}\Delta \Phi_{w}\right)}_{i,j+1/2,k}^{n+1}-{\left(T\lambda_{w}\Delta \Phi_{w}\right)}_{i,j-1/2,k}^{n+1}\\ \;+{\left(T\lambda_{w}\Delta \Phi_{w}\right)}_{i,j,k+1/2}^{n+1}-{\left(T\lambda_{w}\Delta \Phi_{w}\right)}_{i,j,k-1/2}^{n+1}+{Qw}_{i,j,k}^{n+1}\\ \;=\left[{\left(\frac{v\phi s_{w}}{B_{w}}\right)}_{i,j,k}^{n+1}-{\left(\frac{v\phi s_{w}}{B_{w}}\right)}_{i,j,k}^{n}\right]/\Delta t \end{array}$$
for polymer:
  (27) $$\begin{array}{l} {\left(T\lambda_{w}c_{p}\Delta \Phi_{w}\right)}_{i+1/2,j,k}^{n+1}-{\left(T\lambda_{w}c_{p}\Delta \Phi_{w}\right)}_{i-1/2,j,k}^{n+1}+{\left(T\lambda_{w}c_{p}\Delta \Phi_{w}\right)}_{i,j+1/2,k}^{n+1}-{\left(T\lambda_{w}c_{p}\Delta \Phi_{w}\right)}_{i,j-1/2,k}^{n+1}\\ \;+{\left(T\lambda_{w}c_{p}\Delta \Phi_{w}\right)}_{i,j,k+1/2}^{n+1}-{\left(T\lambda_{w}c_{p}\Delta \Phi_{w}\right)}_{i,j,k-1/2}^{n+1}+{\left(Q_{w}^{}c_{p}\right)}_{i,j,k}^{n+1}\\ \;=\{{\left[\frac{v\phi (1-f_{ipv})s_{w}c_{p}}{B_{w}}\right]}_{i,j,k}^{n+1}-{\left[\frac{v\phi (1-f_{ipv})s_{w}c_{p}}{B_{w}}\right]}_{i,j,k}^{n}\\ \;+{\left[v(1-f_{ipv})(1-\phi )\rho_{r}c_{ap}\right]}_{i,j,k}^{n+1}-{\left[v(1-f_{ipv})(1-\phi )\rho_{r}c_{ap}\right]}_{i,j,k}^{n}\}/\Delta t \end{array}$$
for oil:
  (28) $$\begin{array}{l} {\left(T\lambda_{o}\Delta \Psi_{o}\right)}_{i+1/2,j,k}^{n+1}-{\left(T\lambda_{o}\Delta \Psi_{o}\right)}_{i-1/2,j,k}^{n+1}+{\left(T\lambda_{o}\Delta \Psi_{o}\right)}_{i,j+1/2,k}^{n+1}-{\left(T\lambda_{o}\Delta \Psi_{o}\right)}_{i,j-1/2,k}^{n+1}\\ \;+{\left(T\lambda_{o}\Delta \Psi_{o}\right)}_{i,j,k+1/2}^{n+1}-{\left(T\lambda_{o}\Delta \Psi_{o}\right)}_{i,j,k-1/2}^{n+1}+{Qo}_{i,j,k}^{n+1}\\ \;=\left[{\left(\frac{v\phi s_{o}}{B_{o}}\right)}_{i,j,k}^{n+1}-{\left(\frac{v\phi s_{o}}{B_{o}}\right)}_{i,j,k}^{n}\right]/\Delta t \end{array}$$

where n and (i,j,k) are the time step number and the grid block number. $T_{i+1/2,j,k}=\frac{2{\left(dydzk_{xx}\right)}_{i,j,k}{\left(dydzk_{xx}\right)}_{i+1,j,k}}{{\left(dydzk_{xx}\right)}_{i,j,k}dx_{i+1,j,k}+{\left(dydzk_{xx}\right)}_{i+1,j,k}dx_{i,j,k}}$, which is the conductivity coefficient in the *x* direction between the grid blocks (i,j,k) and (i+1,j,k) in m^2^·m, in which dx,dy, and dz are the sizes of the grid block in the *x*, *y* and *z* directions in m, respectively; and $k_{xx}$ is the absolute permeability in the *x* direction. $\Delta \Phi_{wi+1/2,j,k}=p_{wi+1,j,k}-p_{wi,j,k}-\frac{1}{2}\left(\rho_{wi+1,j,k}+\rho_{wi,j,k}\right)g\left(D_{i+1,j,k}-D_{i,j,k}\right)$ is in Pa, and

$$\lambda_{wi+1/2,j,k}=\{\begin{array}{c} {\left(\frac{k_{rw}}{\mu_{we}B_{w}R_{k}}\right)}_{i+1,j,k}\;if\;\Delta \Phi_{wi+1/2,j,k}\geq 0\\ {\left(\frac{k_{rw}}{\mu_{we}B_{w}R_{k}}\right)}_{i+1,j,k}\;if\;\Delta \Phi_{wi+1/2,j,k}<0 \end{array}$$

is in (Pa·s)^−1^.

$$\Delta \Psi_{oi+1/2,j,k}=\{\begin{array}{c} \Delta \Phi_{0i+1/2,j,k}-{\left(Gd_{x}\right)}_{i+1/2,j,k}\;if\;\Delta \Phi_{0i+1/2,j,k}>{\left(Gd_{x}\right)}_{i+1/2,j,k}\\ 0\;if\;\Delta \Phi_{0i+1/2,j,k}\leq {\left(Gd_{x}\right)}_{i+1/2,j,k} \end{array}$$

is in Pa, where $\Delta \Phi_{oi+1/2,j,k}=p_{oi+1,j,k}-p_{oi,j,k}-\frac{1}{2}\left(\rho_{oi+1,j,k}+\rho_{oi,j,k}\right)g\left(D_{i+1,j,k}-D_{i,j,k}\right)$ is in Pa, and ${\left(Gd_{x}\right)}_{i+1/2,j,k}=\frac{1}{2}\left(G_{i+1,j,k}dx_{i+1,j,k}+G_{i,j,k}dx_{i,j,k}\right)$, which is in Pa. $Q_{wi,j,k}$ and $Q_{oi,j,k}$ are the water and oil flow rates in the grid block (i,j,k) under the ground standard conditions in m^3^/s, respectively. Production is negative, and injection is positive. $v_{i,j,k}$ is the volume of the grid block (i,j,k). Other similar quantities can be obtained but are not indicated here.

Equations (26)–(28) can form a system of nonlinear equations. To solve it and ensure stability during the calculation process, the full implicit method was applied. For the solution of each specific time step, the Newton-Raphson method, an iterative method, was applied. More details can be seen in Chen’s research [50,51]. Finally, pressure distributions, water and oil phase saturation distributions, polymer saturation distributions and production indicators were obtained.

## 3. Results and Discussion

### 3.1. Rheology of Polymer Solution and Heavy Oil

**Figure 4:**
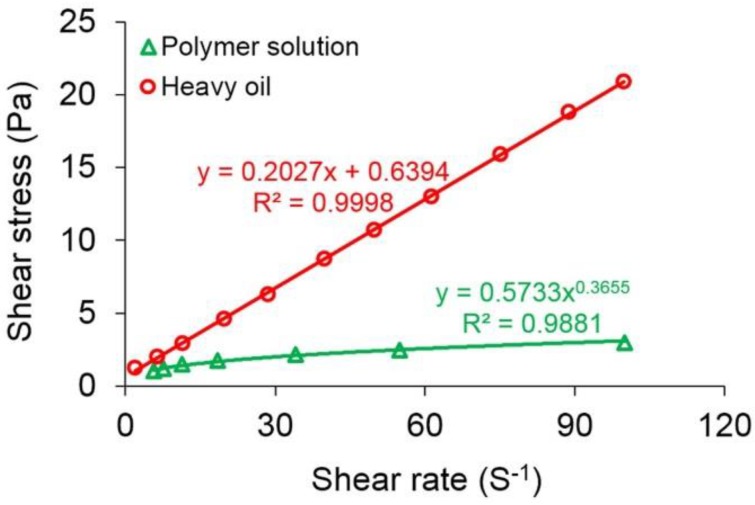
The rheological charts of the polymer solution with a concentration of 2000 mg/L and the original heavy oil.

The rheological charts of the polymer solution with a concentration of 2000 mg/L and the original heavy oil are presented in Figure 4. From the plot of the polymer solution, the shear stress and shear rate show a good power law relationship, and the square of the correlation coefficient (*R*^2^) reaches 0.9881. Their relationship expression can be written as:

(29) $$\tau_{p}=0.5733{\dot{\gamma }}_{p}^{0.3655}$$

where $\tau_{p}$ and ${\dot{\gamma }}_{p}$ are the shear stress and shear rate of the polymer solution in Pa and s^−1^, respectively. The flow behavior index of the polymer solution is 0.3655, less than 1, showing a typical shear thinning. From the plot of the original heavy oil, the relationship between the shear stress and shear rate is linear, but the line with the *R*^2^ of up to 0.9998 does not pass through the origin of the coordinate axes. Their relationship expression can be given as:

(30) $$\tau_{o}=0.2027{\dot{\gamma }}_{o}+0.6394$$

where $\tau_{o}$ and ${\dot{\gamma }}_{o}$ are the shear stress and shear rate of the heavy oil in Pa and s^−1^, respectively. It illustrates that the heavy oil has a typical Bingham fluid rheological feature [52,53].

**Figure 5:**
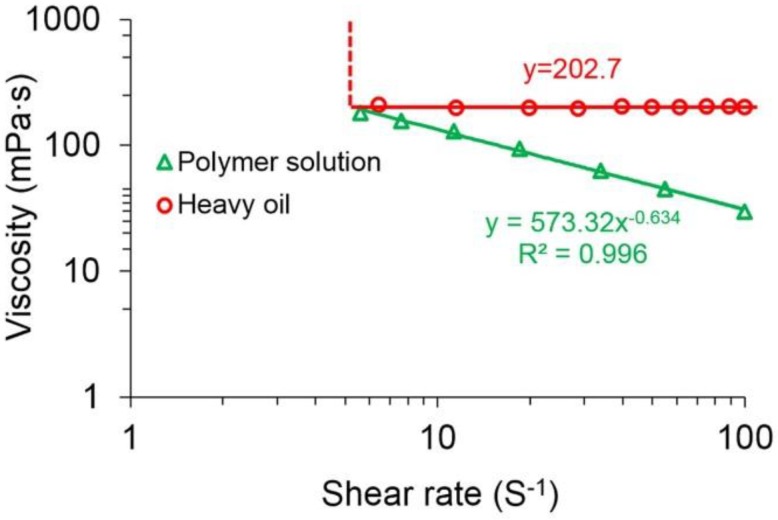
The viscosities of the polymer solution with a concentration of 2000 mg/L and the original heavy oil at different shear rates.

The viscosities of the polymer solution with a concentration of 2000 mg/L and the original heavy oil at different shear rates are indicated in Figure 5. From the plot of the polymer solution, an excellent power law relationship between the viscosity and shear rate is obvious. The relationship expression with the *R*^2^ of up to 0.996 can be expressed as follows:

(31) $$\mu_{p}=573.32{\dot{\gamma }}_{p}^{-0.634}$$

where $\mu_{p}$ is the viscosity of the polymer solution in mPa·s. It also shows that the polymer solution exhibits significant shear thinning rheological properties. The viscosity of the polymer solution greatly decreases with an increase in shear rate. Its viscosity at 100 s^−1^ is 29.57 mPa·s, which is about one-sixth that at 5.6 s^−1^. The main reason for the shear thinning of the polymer solution is that the entanglements between the polymer molecules are destroyed at a higher shear rate, resulting in a decrease in hydrodynamic radius and a reduction in the viscosity of the polymer solution [54,55]. The plot of the heavy oil shows that this oil has an infinite viscosity at low shear rates, but the viscosity of the heavy oil remains at around 202.7 mPa·s as the shear rate increases. It conforms well to the Bingham fluid characteristics [56]. This experiment also reveals that the selected heavy oil is a Bingham fluid. The mechanism of the heavy oil exhibiting Bingham fluid properties becomes evident when its network structure becomes much like a solid and only elastically deforms without flowing under low stress conditions [57]. However, when the external force exceeds the yield stress, its network structure will be destroyed and then it will flow.

**Figure 6:**
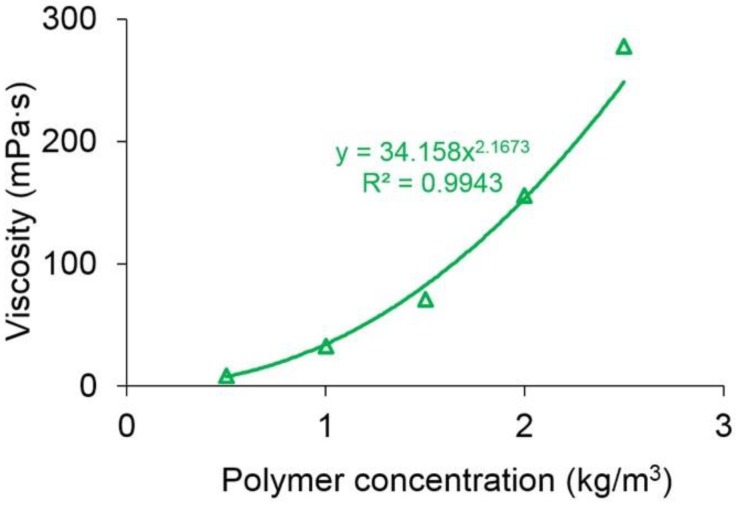
The relationship between the viscosity of the polymer solution and polymer concentration.

Figure 6 shows the relationship between the viscosity of the polymer solution and polymer concentration. Obviously, they have a good power law relationship. The relationship expression with an *R*^2^ of up to 0.9943 is:

(32) $$\mu_{p}=34.158c_{p}^{2.1673}$$

Different from the relationship between polymer solution viscosity and shear rate, the viscosity of the polymer solution significantly increases with an increase in polymer concentration. The viscosity of the polymer solution with a concentration of 2500 mg/L is 278 mPa·s, which is about 34 times greater than that with a concentration of 500 mg/L. The main reason for this finding is that the longer molecular chains and more entanglements are in the polymer solution with a higher polymer concentration, resulting in a larger hydrodynamic radius, which increases the viscosity of the polymer solution [58].

### 3.2. Threshold Pressure Gradient of Heavy Oil

**Figure 7:**
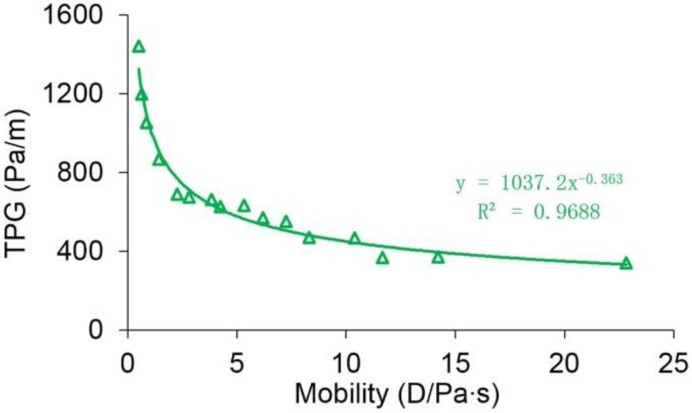
The relationship between the TPG and mobility.

The measured TPG of heavy oil is given in Table 6. Table 6 clearly shows that the TPG increases with an increase in the viscosity of heavy oil under the same permeability conditions, and with a decrease in permeability under the same viscosity of heavy oil conditions. This is attributed to the fact that the flow of heavy oil through the rock is mainly determined by the properties of heavy oil and rock [59]. In the case of the same rock conditions, the heavy oil with a higher viscosity has a stronger network structure, which means that more force is needed to deform it before flow occurs. For the same heavy oil, a higher resistance will appear when it flows through the rock with a lower permeability, and a greater force is required to overcome the higher resistance to make it flow. With consideration of both the effects of the viscosity of the heavy oil and permeability of the core on the TPG of heavy oil, the mobility is introduced to compute the TPG of heavy oil, which is:

(33) $$M=\frac{k_{r}}{\mu_{o}}$$

where M is the mobility in D/Pa·s, and $k_{r}$ is the permeability of rock in D. The relationship between the TPG and mobility is provided in Figure 7. Obviously, they have an excellent power-law relation, which is expressed when the *R*^2^ of 0.9688 is:

(34) $$G=1037.2M^{-0.363}$$

It can be also seen from Figure 7 that the TPG decreases with an increase in mobility. The TPG of the heavy oil when the mobility is 22.83 D/Pa·s was 342.32 Pa/m, which is approximately one-fourth that when the mobility is 0.51 D/Pa·s.

### 3.3. Numerical Simulation

#### 3.3.1. Validation

**Figure 8:**
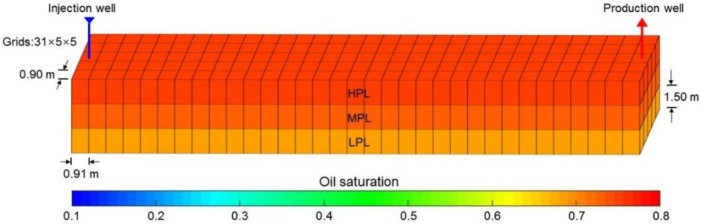
The grid system, well location and 3D distributions of initial oil saturation of Case 1.

**Figure 9:**
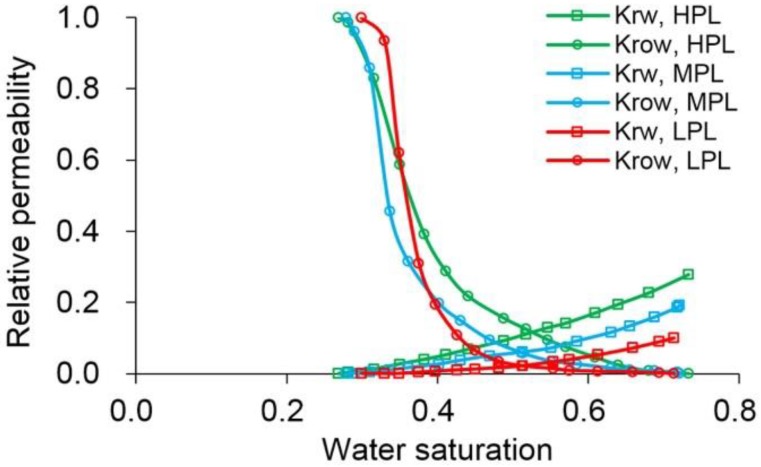
The relative permeabilities used in Case 1.

**Figure 10:**
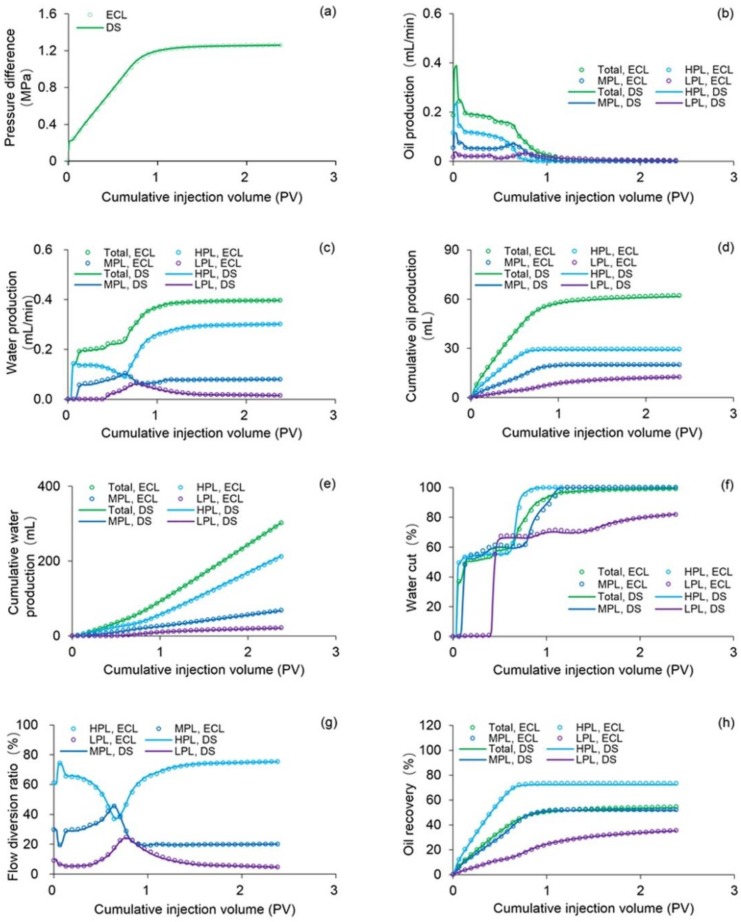
Comparison results of (**a**) pressure difference, (**b**) oil production, (**c**) water production, (**d**) cumulative oil production, (**e**) cumulative water production, (**f**) water cut, (**g**) flow diversion ratio and (**h**) oil recovery of Case 1 simulated by the ECLIPSE V2013.1 software and the designed simulator.

**Figure 11:**
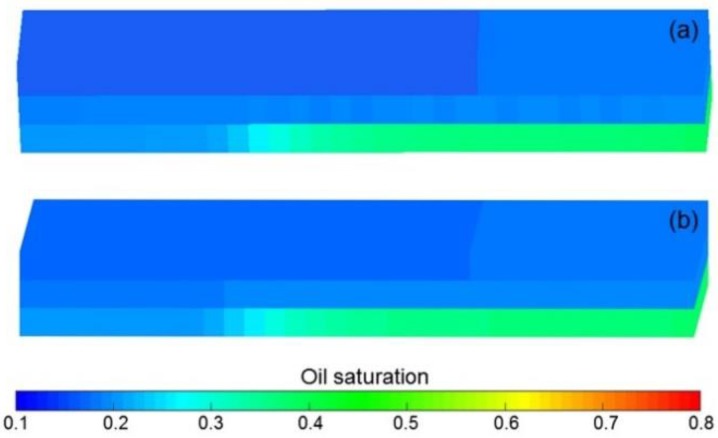
Comparison of 3D remaining oil saturation distributions after cumulative injection volume of 2.4 PV based on (**a**) ECLIPSE V2013.1 software and (**b**) the designed simulator for running Case 1.

The simulation results of Case 1 run by the ECLIPSE V2013.1 software were used to compare Case 1 run by our designed simulator to validate the designed simulator without consideration of TPG because it is a recognized commercial numerical reservoir simulator, and its simulation results are authoritative [15]. The main parameters of Case 1, including the reservoir property, fluid property, initial conditions, production data, grid system, well location, 3D distributions of initial oil saturation and relative permeabilities are given in Table 7, and Figure 8 and Figure 9, where these physical parameters, initial conditions and well location are the same as those in the TPF experiment. Notably, the production data of Case 1 is different from that of the TPF experiment, since only the polymer solution is injected in Case 1, which is a pure polymer flooding (PPF). Figure 10 presents the comparison results of production indicators including pressure differences, oil production, water production, cumulative oil production, cumulative water production, flow diversion ratio, water cut and oil recovery of Case 1 simulated by the ECLIPSE V2013.1 software and designed simulator, where ECL represents the ECLIPSE V2013.1 software and DS represents the designed simulator. Obviously, the simulation results are very close, and the difference of each production indicator is less than 0.4%. Figure 11 indicates the 3D remaining oil saturation distributions after a cumulative injection volume of 2.4 PV of the ECLIPSE V2013.1 software and the designed simulator in running Case 1. The overall remaining oil saturation distributions monitored by the ECLIPSE V2013.1 software and designed simulator are also similar. Thus, the validation of the designed simulator without considering TPG is confirmed, demonstrating high accuracy.

**Figure 12:**
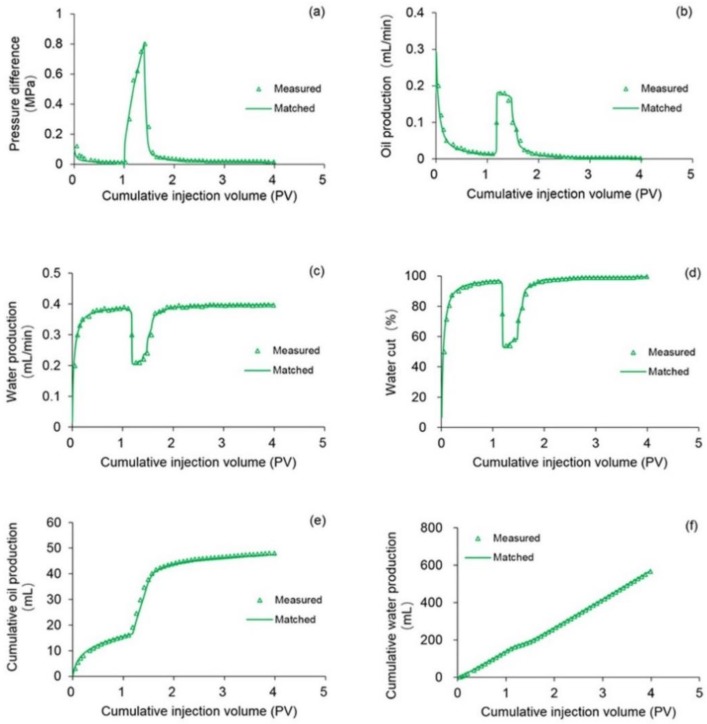
Comparison results of (**a**) pressure difference, (**b**) oil production, (**c**) water production, (**d**) water cut, (**e**) cumulative oil production and (**f**) cumulative water production of Case 2 simulated by the designed simulator and the TPF experiment.

Different from the validation without considering TPG, the validation with considering TPG cannot be conducted by comparing the simulation results of the designed simulator with those of the ECLIPSE V2013.1 software because no widely accepted commercial numerical reservoir simulation software including ECLIPSE V2013.1 software considers TPG [29,35]. To validate the designed simulator with TPG consideration, the data of an actual typical polymer flooding (TPF) experiment was used to compare with its simulation results run by the designed simulator, which was marked as Case 2. The parameters including the physical parameters, initial conditions and well location of Case 2 were the same as those in Case 1, and the different parameters of Case 2 when compared with Case 1 are provided in Table 8. Figure 12 illustrates the comparison results of pressure difference, oil production, water production, water cut, cumulative oil production, and cumulative water production of Case 2 simulated by the designed simulator and the TPF experiment. The simulation results of the designed simulator are in good agreement with the TPF experimental results, and the difference of each production indicator is less than 1.8%. Thus, the validation performed with TPG is positive and acceptable.

#### 3.3.2. Effect of Polymer Solution Shear Thinning

The flow behavior index characterizes the extent to which the fluid deviates from Newtonian fluid [60] and can be used to describe the degree of polymer solution shear thinning. Therefore, Cases 3–9 were conducted to analyze the effect of polymer solution shear thinning on the four main production indicators including pressure difference, water cut, LPL flow diversion ratio and oil recovery of both the PPF and TPF, where the parameters of Cases 3–5 were the same as Case 1 with the exception of the polymer solution flow behavior indexes of 0, 0.24 and 0.18, respectively; the parameters of Cases 6, 8 and 9 were the same as Case 2 except for the TPGs of 0 Pa/m and the polymer solution flow behavior indexes of 1, 0.24 and 0.18, respectively; and the parameters of Case 7 were the same as Case 2 except the TPG of 0 Pa/m.

**Figure 13:**
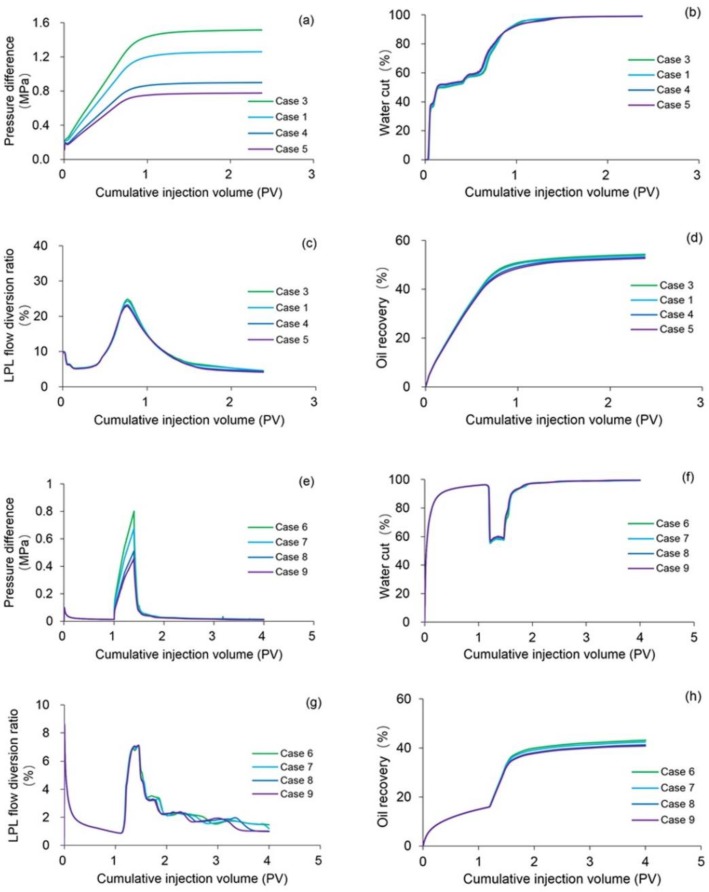
Comparison results of (**a**) pressure difference, (**b**) water cut, (**c**) LPL flow diversion ratio and (**d**) oil recovery of Cases 1 and 3–5, and those of (**e**) pressure difference, (**f**) water cut, (**g**) LPL flow diversion ratio and (**h**) oil recovery of Cases 6–9.

**Figure 14:**
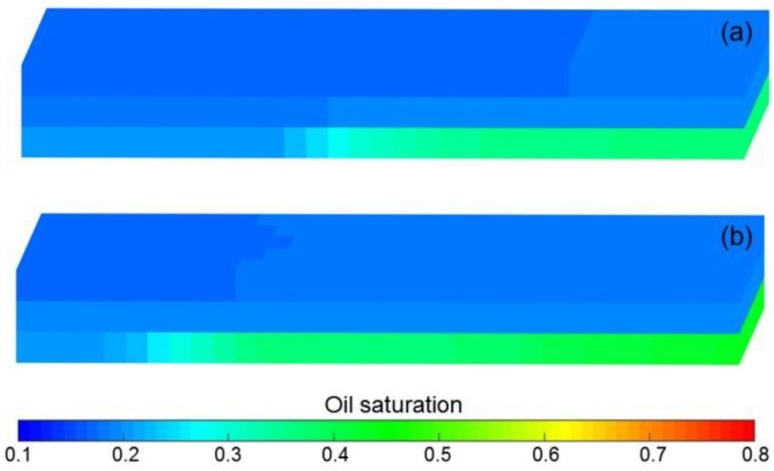
3D remaining oil saturation distributions after polymer flooding of (**a**) Case 3 and (**b**) Case 5.

Figure 13a–d shows the comparison results of pressure difference, water cut, LPL flow diversion ratio and oil recovery of Cases 1 and Cases 3–5. Clearly, the decreases in pressure difference, LPL flow diversion ratio and oil recovery, and an increase in water cut appear with reduction in the polymer solution flow behavior index. Moreover, the production indicator reductions of Cases 1, 4 and 5 vs. Case 3 can be seen in Table 9. In addition, Figure 14 displays the 3D remaining oil saturation distributions after polymer flooding of Case 5. By comparing Figure 14a,b, more oil remains in Case 5. These results are mainly due to the reduction in the viscosity of the polymer solution, caused by its shear thinning, which led to the increase in the water–oil phase mobility ratio, resulting in the unsatisfactory PPF efficiency and more oil remaining in the reservoir after the PPF. The polymer solution shear thinning has a negative effect on the PPF.

**Figure 15:**
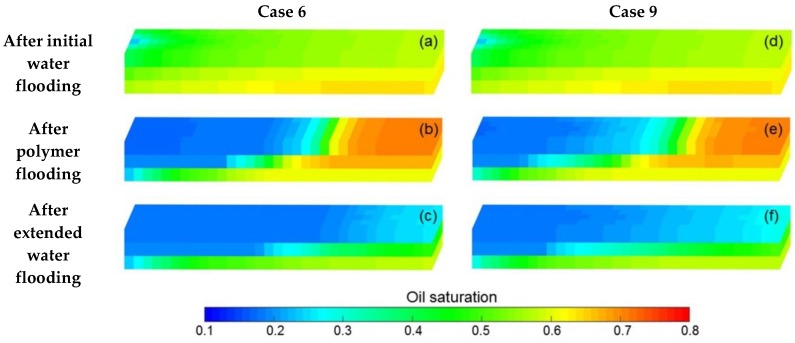
3D remaining oil saturation distributions after (**a**) initial water flooding, (**b**) polymer flooding and (**c**) extended water flooding of Case 6, and those results after (**d**) initial water flooding, (**e**) polymer flooding and (**f**) extended water flooding of Case 9.

The comparison results of pressure difference, water cut, LPL flow diversion ratio, and oil recovery of Cases 6–9, the production indicator reductions of Cases 7–9 vs. Case 6, and 3D remaining oil saturation distributions after initial water flooding, polymer flooding and extended water flooding of Cases 6 and 9 are illustrated in Figure 13e–h, Table 9 and Figure 15, respectively. Obviously, the production indicators after the initial water flooding of Cases 6–9 are the same, and the 3D remaining oil saturation distributions after initial water flooding of Cases 6 and 9 are no different. These are due to the absence of a polymer solution in the initial water flooding, and the polymer solution shear thinning, which has no effect on the simulation results during the initial water flooding. In addition, they also reflect the computational stability and reliability of our designed simulator. However, after the polymer flooding as well as the extended water flooding, the decreases in pressure difference, the LPL flow diversion ratio and oil recovery, and an increase in water cut occur with a decrease in the polymer solution flow behavior index, and the remaining oil saturation of Case 9 is more than that of Case 6. These occurrences can be also attributed to the reduction in the viscosity of the polymer solution, which is followed by an increase in the water-oil phase mobility ratio, resulting in the disappointing TPF efficiency and more oil remaining in the reservoir after the TPF. The polymer solution shear thinning also had an adverse effect on the TPF.

In summary, the polymer solutions shear thinning has a disadvantageous impact on the polymer flooding including the PPF and TPF in the heavy oil reservoirs, and the effect of polymer solution shear thinning on the PPF is less than that on the TPF after comparing the simulation results of these specific cases.

#### 3.3.3. Effect of Heavy Oil Threshold Pressure Gradient

In order to investigate the effect of heavy oil TPG on the four main production indicators—pressure difference, water cut, LPL flow diversion ratio and oil recovery of polymer flooding including both the PPF and TPF, Cases 10–15 were carried out, where the parameters of Cases 10–12 were the same as Case 3 except that their heavy oil TPGs were 5, 10 and 20 times that of Case 3, respectively. The parameters of Cases 13–15 were the same as Case 6 except that their heavy oil TPGs were 5, 10 and 20 times that of Case 6, respectively.

**Figure 16:**
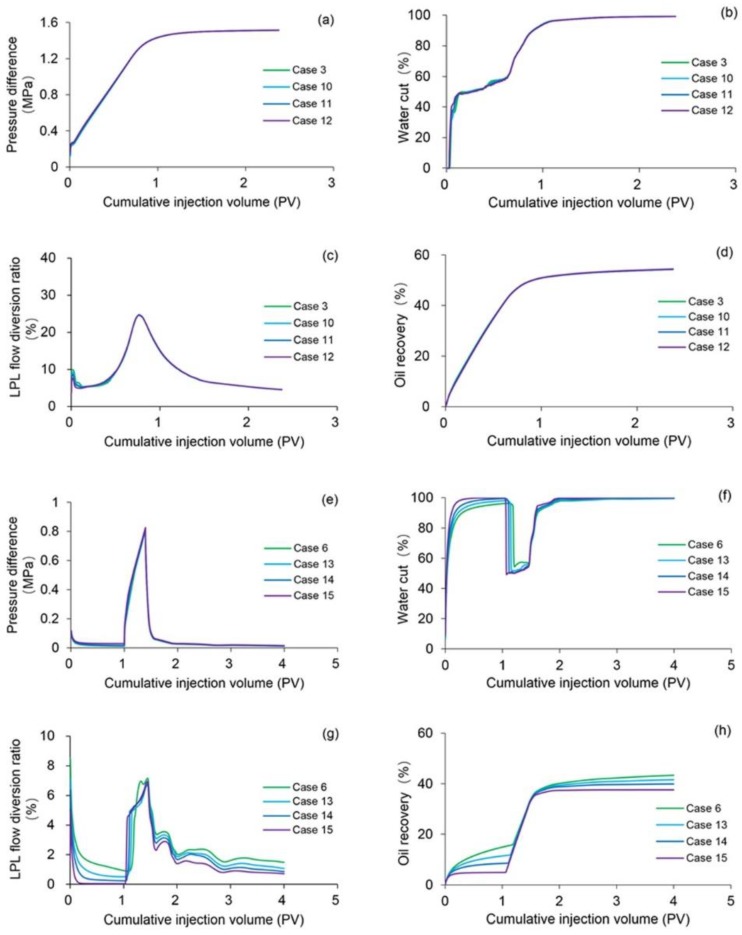
Comparison results of (**a**) pressure difference, (**b**) water cut, (**c**) LPL flow diversion ratio and (**d**) oil recovery of Cases 3 and 10–12, and those of (**e**) pressure difference, (**f**) water cut, (**g**) LPL flow diversion ratio, and (**h**) oil recovery of Cases 6 and 13–15.

**Figure 17:**
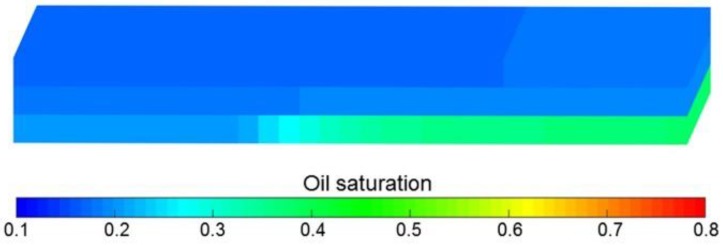
3D remaining oil saturation distributions after polymer flooding of Case 12.

Figure 16a–d demonstrates the comparison results of pressure difference, water cut, LPL flow diversion ratio and oil recovery of Cases 3 and 10–12. Obviously, the pressure difference and water cut increased, and the LPL flow diversion ratio and oil recovery decrease with an increase in heavy oil TPG. Moreover, the production indicator reductions of Cases 10–12 vs. Case 3 are presented in Table 10, and their absolute values are relatively small. In addition, Figure 17 indicates the 3D remaining oil saturation distributions after polymer flooding of Case 12. By comparing Figure 17 and Figure 14a, although the difference between the oil saturation distributions of Cases 12 and 3 are inconspicuous, the oil saturation of Case 12 is still slightly greater than that of Case 3. The reason for these results is that the TPG made heavy oil flow difficult in the reservoir, resulting in more remaining oil after the PPF and unsatisfactory PPF efficiency. The heavy oil TPG has a negative effect on the PPF but not significantly.

**Figure 18:**
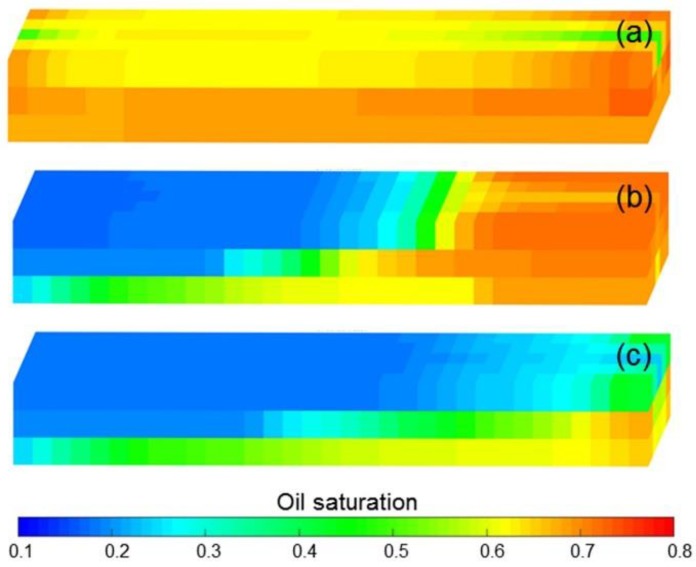
3D remaining oil saturation distributions after (**a**) initial water flooding, (**b**) polymer flooding and (**c**) extended water flooding of Case 15.

The comparison results of pressure difference, water cut, LPL flow diversion ratio and oil recovery of Cases 6 and 13–15; the production indicator reductions of Cases 13–15 vs. Case 6, and 3D remaining oil saturation distributions after initial water flooding, polymer flooding and extended water flooding of Cases 6 and 13–15 are demonstrated in Figure 16e–h, Table 10 and Figure 18, respectively. Evidently, the pressure difference and water cut increase, and the LPL flow diversion ratio and oil recovery decrease as the heavy oil TPG increases after the initial water flooding. Moreover, the absolute values of the production indicator reductions of Cases 13–15 vs. Case 6 are relatively large. In addition, by comparing Figure 18 and Figure 15d–f, the oil saturation of Case 15 is distinctly more than that of Case 6. The major reason for these results is that the heavy oil flow became difficult due to the existence of TPG, which is followed by more oil remaining in the reservoir after the initial water flooding and the disappointing initial water flooding performance. The heavy oil TPG has a serious adverse impact on the initial water flooding. Different from the results after the initial water flooding, after the polymer flooding, only the pressure difference increases as the heavy oil TPG grew; meanwhile the water cut, LPL flow diversion ratio and oil recovery reduce with an increase in heavy oil TPG. Moreover, the LPL flow diversion ratio and oil recovery reductions after the polymer flooding are less than those after the initial water flooding. In addition, the polymer flooding EOR under higher TPG conditions is greater than that under lower TPG conditions, and the polymer EOR of Case 15 is approximately 9% more than that of Case 6. A better polymer EOR is obtained under the higher heavy oil TPG conditions, and the great significance of polymer flooding for heavy oil reservoirs with the TPG is manifested. However, the oil saturation after both the water flooding and polymer flooding of Case 15 is still higher than that of Case 6. The heavy oil TPG has a negative effect on the oil recovery after both the initial water flooding and polymer flooding. However, after the extended water flooding, the increase in pressure difference and water cut, and the decreases in the LPL flow diversion ratio and oil recovery present as the heavy oil TPG increased, and the remaining oil saturation of Case 15 is more than that of Case 6, which are like those after the initial water flooding. The reason for these results is also the difficulty in heavy oil flow caused by the TPG, leading to more remaining oil in the reservoir and the unsatisfied TPF performance. The heavy oil TPG also has an adverse effect on the TPF.

In summary, the heavy oil TPG has a detrimental impact on the polymer flooding including the PPF and TPF in the heavy oil reservoirs, and the effect of heavy oil TPG on the PPF is much less than that on the TPF based on comparisons of the simulation results of these specific cases. Additionally, the effect of heavy oil TPG on the PPF is also less than that of polymer solution shear thinning on the PPF, but the effect of heavy oil TPG on the TPF is more than that of polymer solution shear thinning on the TPF as noted in the comparison of simulation results in this paper.

#### 3.3.4. Combined Effect of Shear Thinning and Threshold Pressure Gradient

Cases 16 and 17 were conducted to analyze the combined effect of TPG and shear thinning on the four main production indicators including pressure difference, water cut, LPL flow diversion ratio and oil recovery of polymer flooding including both the PPF and TPF, where the parameters of Cases 16 were the same as Case 3 except for the polymer solution flow behavior indexes of 0.18 and the fact that its heavy oil TPG was 20 times that of Case 3, and the parameters of Cases 17 were the same as Case 6 with the exception of the polymer solution flow behavior indexes of 0.18 with a heavy oil TPG 20 times greater than that of Case 3.

**Figure 19:**
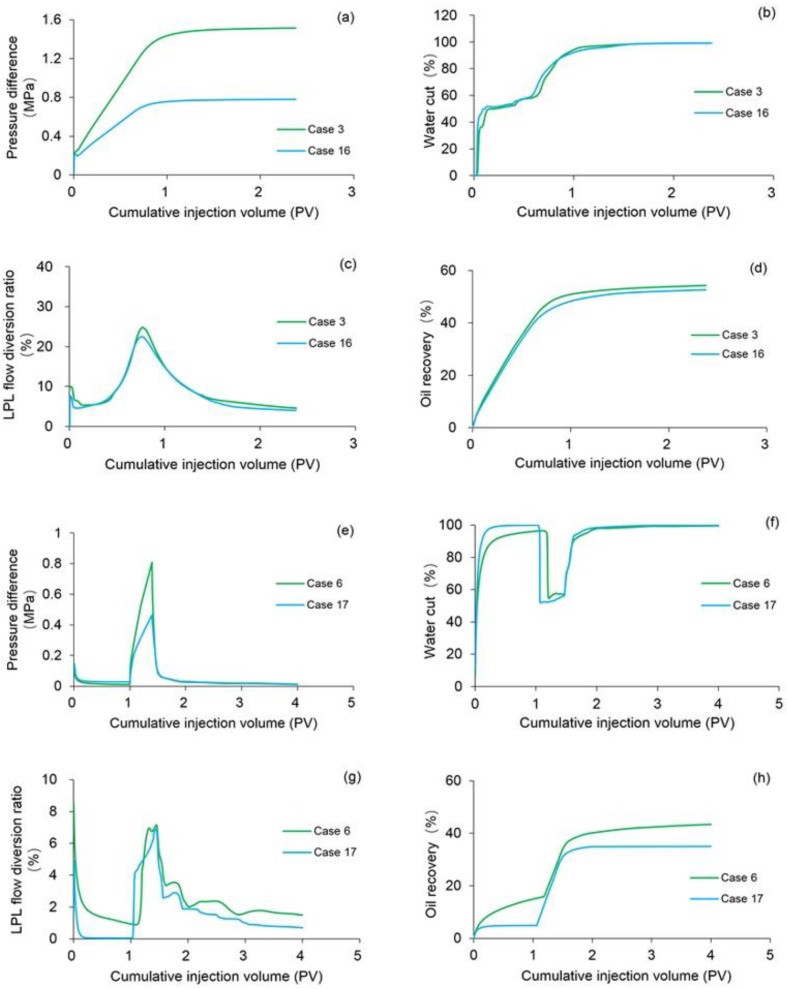
Comparison results of (**a**) pressure difference, (**b**) water cut, (**c**) LPL flow diversion ratio and (**d**) oil recovery of Cases 3 and 16, and those of (**e**) pressure difference, (**f**) water cut, (**g**) LPL flow diversion ratio and (**h**) oil recovery of Cases 6 and 17.

**Figure 20:**
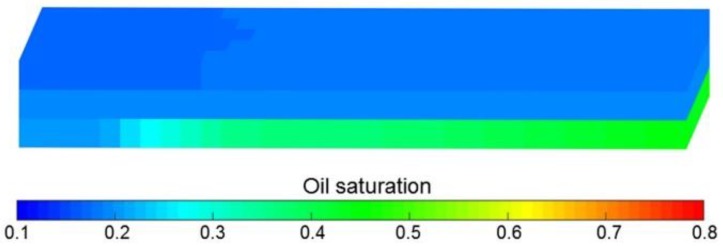
3D remaining oil saturation distributions after polymer flooding of Case 16.

Figure 19a–d compares pressure difference, water cut, LPL flow diversion ratio and oil recovery of Cases 3 and 16. By comparing the production indictors of Case 16 with those of Case 3, decreases in pressure difference, LPL flow diversion ratio and oil recovery, and an increase in water cut occur. Moreover, the production indicator reductions of Case 16 are shown in Table 11. In addition, the 3D remaining oil saturation distributions after polymer flooding of Case 16 is displayed in Figure 20. By comparing Figure 20 and Figure 14a, more remaining oil is in Case 16. The comparison results of Case 16 vs. Case 3 are like those of Cases 1, 4 and 5 vs. Case 3. This is attributed to the fact that the effect of polymer solution shear thinning is greater than that of heavy oil TPG on the PPF in this specific study. According to the comparison results, the combined effects of both polymer solution shear thinning and heavy oil TPG on PPF are negative.

**Figure 21:**
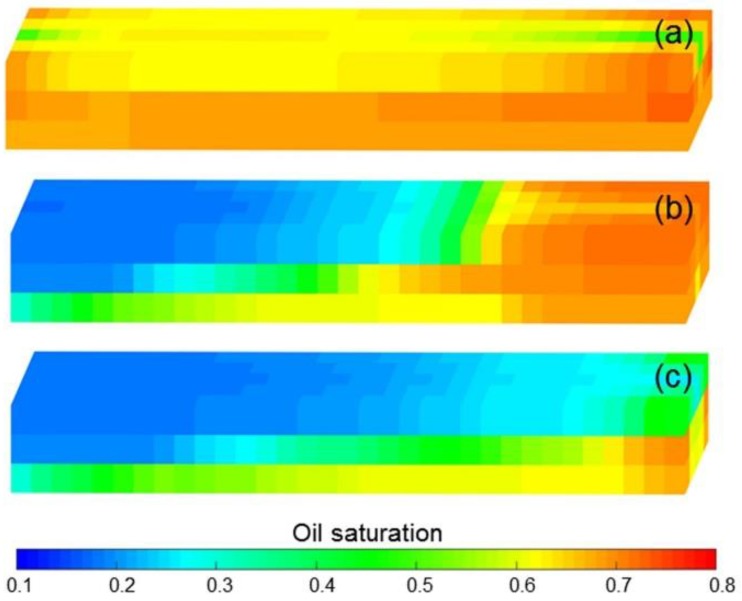
3D remaining oil saturation distributions after (**a**) initial water flooding, (**b**) polymer flooding and (**c**) extended water flooding of Case 17.

The comparison results of pressure difference, water cut, LPL flow diversion ratio and oil recovery of Cases 6 and 17, the production indicator reductions of Case 17 vs. Case 6, and 3D remaining oil saturation distributions after initial water flooding, and polymer flooding and extended water flooding of Cases 6 and 17 are presented in Figure 19e–h, Table 11 and Figure 21, respectively. Obviously, the simulation results of Case 17 are the same as those of Case 15 during the initial water flooding since no polymer solution is involved in the initial water flooding and only the effect of heavy oil TPG, such as the comparison results after the initial water flooding of Case 6 and 17 are no longer specified here. After the polymer flooding, all four production indictors increase; the remaining oil saturation of Case 17 is higher than that of Case 6. The combined effects of both polymer solution shear thinning and heavy oil TPG on the oil recovery after both the initial water flooding and polymer flooding is also negative. After the extended water flooding, the increase in pressure difference and water cut and the decrease in the LPL flow diversion ratio and oil recovery took place, and the remaining oil saturation of Case 17 is higher than that of Case 6. These comparison results are like those of Cases 13–15 vs. Case 6 because the effect of the heavy oil TPG is dominant. The combined effects of both polymer solution shear thinning and heavy oil TPG on PPF are also negative.

In summary, the combined effects of both polymer solution shear thinning and heavy oil are adverse on the polymer flooding, including the PPF and TPF in the heavy oil reservoirs, and the combined effect is greater than the single effect by comparing the simulation results of these specific cases.

## 4. Conclusions

In this study, physical experiments and numerical simulations were combined with the aim of investigating the effect of non-Newtonian flow on polymer flooding in heavy oil reservoirs and giving theoretical and technical guidance. The physical experiment results showed that the flow of both the polymer solution with a concentration of 2000 mg/L and original heavy oil exhibit the non-Newtonian flow characteristics. The shear stress and shear rate of the polymer solution with a concentration of 2000 mg/L indicates a good power law relationship with the *R*^2^ of 0.9881; its flow behavior index is 0.3655, and its viscosity decreases as shear rate increases, showing a typical shear thinning performance. The shear stress and shear rate of the original heavy oil is linear with the *R*^2^ of up to 0.9998, however, the fitting line does not pass through the origin of the coordinate axes; the viscosity of the original heavy oil is infinite at the low shear rate and remains at an approximate but steady 202.7 mPa·s as the shear rate increases, presenting Bingham fluid characteristics. The heavy oil needs to overcome the TPG to flow, and its TPG increases with a reduction in rock permeability and an increase in heavy oil viscosity. Moreover, the heavy oil TPG and the mobility have an excellent power-law relation as expressed by the *R*^2^ of 0.9688, and the heavy oil TPG increases with a decrease in mobility. Furthermore, the new 3D two-phase polymer flooding simulator considering the non-Newtonian flow was designed and validated with high accuracy and reliability by comparing its simulation results with those performed by the commercial software, as well as with the results obtained from the TPF experiments. Additionally, the effect of non-Newtonian flow on the production indicators was studied using the deigned simulator. According to the simulation results, the oil recoveries of the PPF and TPF, with the polymer solution flow behavior indexes of 0.18, are 1.66% and 2.49% lower than those of the PPF and TPF, with the polymer solution flow behavior index of 1, respectively, demonstrating that the polymer solution shear thinning has a disadvantageous effect on the polymer flooding in the heavy oil reservoirs, and its effect on the PPF was less than that on the TPF. For the heavy oil, considering a TPG of 20 times greater than its original value, the oil recoveries of PPF and TPF are reduced by 0.01% and 5.77%, respectively, illustrating that the heavy oil TPG has a negative effect on the polymer flooding in the heavy oil reservoirs, and its effect on the PPF is slight, less than that on the TPF. However, a better polymer EOR after the polymer flooding of TPF with the greater heavy oil TPG is obtained, strongly proving the importance of polymer flooding for heavy oil reservoirs. In addition, the effect of heavy oil TPG on the PPF is also less than that of polymer solution shear thinning on the PPF, while the effect of heavy oil TPG on the TPF is more than that of polymer solution shear thinning on the TPF. The oil recoveries of the PPF and TPF, with the polymer solution flow behavior indexes of 0.18 and the heavy oil TPGs 20 times the original heavy oil TPG, are 1.74% and 8.35% lower than those of the PPF and TPF, without shear thinning and heavy oil TPF, respectively. To minimize the effects of non-Newtonian flow, including polymer solution shear thinning and heavy oil TPG, on polymer flooding in heavy oil reservoirs, some methods including applying shear resistant polymer and heavy oil TPG reduction methods have been proposed [61,62]. In the future, we will conduct experiments to extend and improve our understanding of this technology to the nanoconfined flow in unconventional reservoirs [63,64] based on the findings presented in this paper.

## Figures and Tables

**Table 1:** Polymer properties.

Properties	Description/Value
Type	HPAM
Molecular weight	2.52 × 10^7^
Solid content, wt %	91
Hydrolysis degree, %	26
Filtration factor	1.4
Dissolution rate, hour	<2
Insoluble matter, wt %	0.15
Granularity ≥ 1.0 mm, %	4.9
Granularity ≤ 0.2 mm, %	2.5

**Table 2:** Ion component concentrations in brine.

Ion Components	Concentration, mg/L
Na^+^ and K^+^	217.73
Ca^2+^	61.16
Mg^2+^	27.66
HCO_3_^−^	309.59
CO_3_^2−^	76.13
SO_4_^2−^	158.35
Cl^−^	135.00
TDS	985.60

**Table 3:** Original heavy oil composition and basic physical properties.

Parameters	Saturate, wt %	Aromatic, wt %	Resin, wt %	Asphaltene, wt %	Density (25 °C), kg/m^3^	Viscosity (25 °C), mPa·s
Value	56.15	28.87	13.52	1.46	938.30	202.70

**Table 4:** Parameters of core samples used to study the relationship between TPG and mobility.

Core Number	Length, cm	Diameter, cm	Porosity, %	Permeability, mD
#1	25.06	2.49	19.42	103
#2	25.10	2.51	19.44	104
#3	25.08	2.50	19.41	103
#4	25.07	2.49	19.44	105
#5	25.18	2.50	21.62	458
#6	25.16	2.51	21.60	456
#7	25.17	2.49	21.60	455
#8	25.16	2.50	21.61	457
#9	25.05	2.50	23.38	863
#10	25.08	2.49	23.40	864
#11	25.02	2.48	23.36	862
#12	25.04	2.49	23.36	862
#13	25.12	2.50	25.04	1683
#14	25.09	2.49	25.08	1685
#15	25.06	2.50	25.12	1686
#16	25.11	2.51	25.10	1685

**Table 5:** Parameters of core samples used to perform polymer flooding experiment.

Parameters	Core Name		
	High Permeability Layer (HPL)	Middle Permeability Layer (MPL)	Low Permeability Layer (LPL)
Length, cm	30	30	30
Width, cm	4.5	4.5	4.5
Height, cm	1.5	1.5	1.5
Porosity, %	26.2	25.6	25
Permeability, mD	1800	900	300

**Table 6:** The measured TPG of heavy oil.

Core Number	Permeability, mD	Oil Sample	Viscosity, mPa·s	TPG, Pa/m
#1	103	#1	202.7	1442.23
#2	104	#2	162.2	1198.81
#3	103	#3	118.7	1055.87
#4	105	#4	73.8	868.76
#5	458	#1	202.7	690.13
#6	456	#2	162.2	675.27
#7	455	#3	118.7	663.83
#8	457	#4	73.8	570.80
#9	863	#1	202.7	627.70
#10	864	#2	162.2	633.98
#11	862	#3	118.7	552.12
#12	862	#4	73.8	368.76
#13	1683	#1	202.7	470.51
#14	1685	#2	162.2	466.65
#15	1686	#3	118.7	370.64
#16	1685	#4	73.8	342.32

**Table 7:** The reservoir property, fluid property, initial conditions and production data of Case 1.

Parameters	Value
Initial porosity in HPL, MPL and LPL, fraction	0.262, 0.256, 0.25
Initial permeability in *x* direction in HPL, MPL and LPL, mD	1800, 900, 300
Initial permeability in *y* direction in HPL, MPL and LPL, mD	1800, 900, 300
Initial permeability in *z* direction in HPL, MPL and LPL, mD	180, 90, 30
Reservoir temperature, °C	25
Rock density in HPL, MPL and LPL, kg/m^3^	2570, 2590, 2610
Rock compressibility in HPL, MPL and LPL, MPa^−1^	2.82 × 10^−3^, 2.78 × 10^−3^, 2.72 × 10^−3^
Stock tank oil density, kg/m^3^	938.3
Initial oil viscosity, mPa·s	202.7
Oil compressibility, MPa^−1^	1.18 × 10^−3^
Oil formation volume factor	1.068
Initial water density, kg/m^3^	1000
Water viscosity, mPa·s	0.69
Water compressibility, MPa^−1^	4.26 × 10^−4^
Water formation volume factor	1.016
Polymer concentration, mg/L	2500
Inaccessible pore volume factor in HPL, MPL and LPL, fraction	0.05, 0.06, 0.08
Maximum polymer absorption in HPL, MPL and LPL, kg/kg	6.88 × 10^−5^, 7.67 × 10^−5^, 8.66 × 10^−5^
Residual resistance factor in HPL, MPL and LPL	2.80, 3.60, 5.20
Initial reservoir pressure, MPa	0
Initial water saturation in HPL, MPL and LPL, fraction	0.24, 0.26, 0.3
Initial oil saturation in HPL, MPL and LPL, fraction	0.76, 0.74, 0.70
Bottom hole pressure of production well, MPa	0
Injected polymer solution during polymer flooding, PV	2.4

**Table 8:** The different parameters of Case 2 when compared with Case 1.

Parameters	Value
TPG in HPL, MPL and LPL, Pa/m	469.45, 603.75, 899.61
Injected water during water flooding, PV	1
Injected polymer solution during polymer flooding, PV	0.4
Injected water during subsequent water flooding after polymer flooding, PV	2.6

**Table 9:** Production indicator reductions of Cases 1, 4 and 5 vs. Case 3, and those of Cases 7–9 vs. Case 6.

Production Indictors		Case Number					
		1	4	5	7	8	9
**After initial water flooding**	Pressure difference, MPa	-	-	-	0.0000	0.0000	0.0000
	Water cut, %	-	-	-	0.0000	0.0000	0.0000
	LPL flow diversion ratio, %	-	-	-	0.0000	0.0000	0.0000
	Oil recovery, %	-	-	-	0.0000	0.0000	0.0000
**After polymer flooding**	Pressure difference, MPa	0.2540	0.6155	0.7371	0.1355	0.2973	0.3529
	Water cut, %	−0.0480	−0.0508	−0.0728	−1.3499	−2.3286	−2.7385
	LPL flow diversion ratio, %	0.0033	0.1814	0.4382	0.0316	0.1320	0.1946
	Oil recovery, %	0.4566	1.2515	1.6614	0.7086	1.0095	1.2173
**After extended water flooding**	Pressure difference, MPa	-	-	-	0.0024	0.0029	0.0029
	Water cut, %	-	-	-	−0.0753	−0.1943	−0.2024
	LPL flow diversion ratio, %	-	-	-	0.2932	0.4682	0.4969
	Oil recovery, %	-	-	-	0.8670	1.9394	2.4850

**Table 10:** Production indicator reductions of Cases 10–12 vs. Case 3, and those of Cases 13–15 vs. Case 6.

Production Indictors		Case Number					
		10	11	12	13	14	15
**After initial water flooding**	Pressure difference, MPa	-	-	-	−0.0013	−0.0048	−0.0167
	Water cut, %	-	-	-	−1.9711	−3.2508	−3.8929
	LPL flow diversion ratio, %	-	-	-	0.4186	0.6880	0.8840
	Oil recovery, %	-	-	-	3.5002	6.4676	10.0672
**After polymer flooding**	Pressure difference, MPa	−0.0001	−0.0002	−0.0003	−0.0041	−0.0083	−0.0167
	Water cut, %	−0.0026	−0.0042	−0.0066	1.5416	3.3325	4.0417
	LPL flow diversion ratio, %	0.0004	0.0031	0.0119	0.4061	0.4852	0.4864
	Oil recovery, %	0.0437	0.0196	0.0141	0.2837	0.5885	0.9841
**After extended water flooding**	Pressure difference, MPa	-	-	-	−0.0001	−0.0005	−0.0015
	Water cut, %	-	-	-	−0.2863	−0.5018	−0.5916
	LPL flow diversion ratio, %	-	-	-	0.4132	0.6257	0.7701
	Oil recovery, %	-	-	-	1.7753	3.4951	5.7741

**Table 11:** Production indicator reductions of Case 16 vs. Case 3, and those of Case 17 vs. Case 6.

Production Indicators		Case Number	
		16	17
**After first water flooding**	Pressure difference, MPa	-	0.0167
	Water cut, %	-	−3.8929
	LPL flow diversion ratio, %	-	0.8840
	Oil recovery, %	-	10.0672
**After polymer flooding**	Pressure difference, MPa	0.7366	0.3443
	Water cut, %	−0.0104	2.1395
	LPL flow diversion ratio, %	0.5591	0.4370
	Oil recovery, %	1.7407	2.0819
**After second water flooding**	Pressure difference, MPa	-	0.0013
	Water cut, %	-	−0.5918
	LPL flow diversion ratio, %	-	0.7901
	Oil recovery, %	-	8.3466
